# Major milestones in translational oncology

**DOI:** 10.1186/s12916-016-0654-y

**Published:** 2016-07-28

**Authors:** Tommaso A. Dragani, Antoni Castells, Vathany Kulasingam, Eleftherios P. Diamandis, Helena Earl, Wade T. Iams, Christine M. Lovly, J. P. Michiel Sedelaar, Jack A. Schalken

**Affiliations:** 1 Fondazione IRCCS Istituto Nazionale dei Tumori, Via G.A. Amadeo 42, I-20133 Milan, Italy; 2 Department of Gastroenterology, Hospital Clinic, University of Barcelona, IDIBAPS, CIBERehd, Barcelona, Catalonia Spain; 3 Department of Laboratory Medicine and Pathobiology, University of Toronto, Toronto, Ontario Canada; 4 Department of Clinical Biochemistry, University Health Network, Toronto, Ontario Canada; 5 Department of Pathology and Laboratory Medicine, Mount Sinai Hospital, Toronto, Ontario Canada; 6 Deptartment of Oncology, University of Cambridge, Cambridge, UK; 7 NIHR Cambridge Biomedical Research Centre, Addenbrooke’s Hospital, Cambridge Biomedical Campus, Cambridge, UK; 8 Department of Medicine Vanderbilt University Medical Center, Nashville, TN USA; 9 Department of Cancer Biology, Vanderbilt University Medical Center, Nashville, TN USA; 10 Vanderbilt-Ingram Cancer Center, Nashville, TN USA; 11 Department of Urology, Radboud University Medical Center, Nijmegen, The Netherlands

**Keywords:** Biomarkers, Cancer vaccines, Early diagnosis, Genetic profile, Immunotherapy, Individual risk, Liquid biopsy, Mutational landscape, Next generation sequencing, Tumorigenesis, Colorectal, Ovarian, Breast, Lung, Prostate

## Abstract

Translational oncology represents a bridge between basic research and clinical practice in cancer medicine. Today, translational research in oncology benefits from an abundance of knowledge resulting from genome-scale studies regarding the molecular pathways involved in tumorigenesis. In this Forum article, we highlight the state of the art of translational oncology in five major cancer types. We illustrate the use of molecular profiling to subtype colorectal cancer for both diagnosis and treatment, and summarize the results of a nationwide screening program for ovarian cancer based on detection of a tumor biomarker in serum. Additionally, we discuss how circulating tumor DNA can be assayed to safely monitor breast cancer over the course of treatment, and report on how therapy with immune checkpoint inhibitors is proving effective in advanced lung cancer. Finally, we summarize efforts to use molecular profiling of prostate cancer biopsy specimens to support treatment decisions. Despite encouraging early successes, we cannot disregard the complex genetics of individual susceptibility to cancer nor the enormous complexity of the somatic changes observed in tumors, which urge particular attention to the development of personalized therapies.

## Introduction

**Fig. 1:**
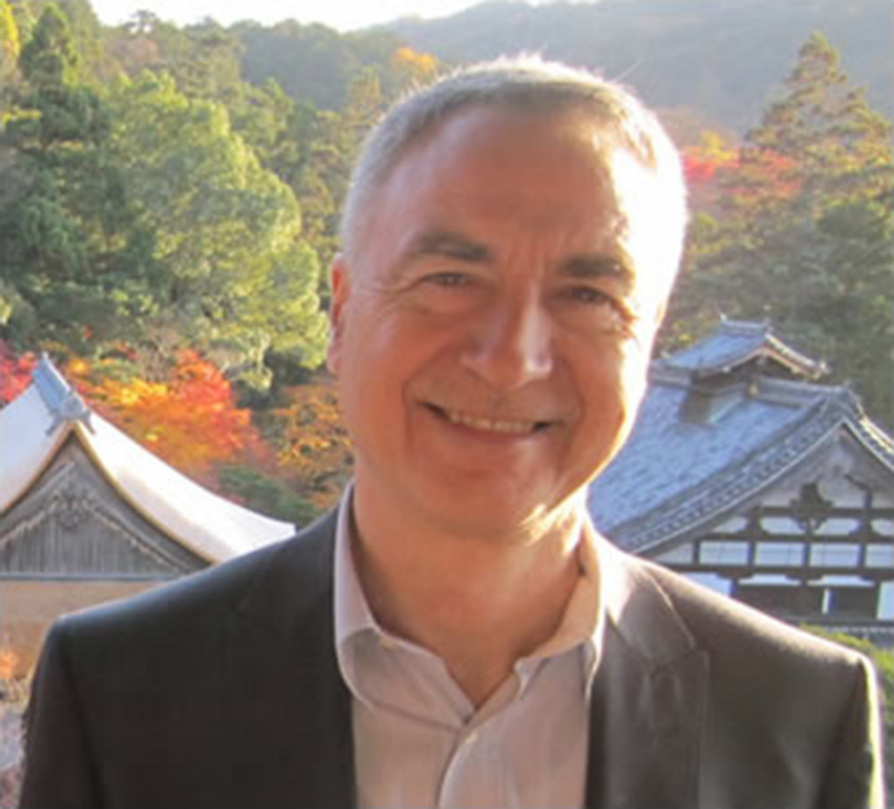
Tommaso A. Dragani. Tommaso A. Dragani is Director of the Research Unit of Genetic Epidemiology and Pharmacogenomics at Fondazione IRCCS Istituto Nazionale dei Tumori in Italy. He has been a pioneer of research on the genetic predisposition to cancer. His early studies in animal models led to the identification and characterization of chromosomal loci that determine the inherited predispositions to liver, lung and skin tumorigenesis. He has since extended this research to humans by carrying out population-based case-control studies to identify genetic polymorphisms associated with the risk for and prognosis of lung cancer, thus contributing to the field of complex (polygenic) genetic predisposition to cancer. Dr. Dragani’s research group discovered the mechanism underlying the association between polymorphisms in nicotinic receptor subunit genes and the risks of both lung cancer and nicotine dependence. Additionally, he led the first genome-wide pharmacogenomic study on pain relief, which discovered that multiple loci modulate the response to opioid therapy for cancer pain

Tommaso A. Dragani (Fig. 1)

The term “translational oncology” first appeared in the scientific literature about 15 years ago [1, 2], although this field has a longer history, as it was the paradigm for translational research in general. The concept of translational research emerged from the US National Cancer Institute at the 1992 National Conference on Cancer Prevention and Early Detection, where James L. Mulshine and colleagues discussed how epithelial cancers could be blocked in the early stages of tumorigenesis if agents were developed to interfere with growth factors or other molecules involved in tumor promotion: ‘*Through this type of translational research, important applications of molecular biology may greatly improve the success of preventative strategies for cancer control*’ [3]. Shortly afterwards, George D. Demetri, from the Dana-Farber Cancer Institute, wrote ‘*research and clinical development of hematopoietic cytokines has been a magnificent example of “bench to bedside” translational research*’ [4].

As translational research established itself as a bridge between basic research and clinical practice, its application spread beyond cancer to disease in general and then to non-biomedical fields such as engineering. This latter development required an update of terminology within the biomedical community. Thus, the term ‘translational medicine’, which had been used occasionally in the 1990s (e.g., [5]), became established in medical discourse upon the 2003 founding of the *Journal of Translational Medicine*. In its wake, dozens of journals on translational research were founded in a myriad of disciplines of biomedicine, including cancer research, for which it acquired a new term – translational oncology. While this field has been the pathfinder for translational research over the past 25 years, there has been ‘*a remarkable acceleration in the pace of translational cancer medicine*’ in the past decade thanks to the availability of powerful ‘*molecular characterization technologies*’ [6].

One exciting focus of translational oncology today is immunotherapy, where the combination of basic research on tumor cell biology and technological advances in immune cell engineering is permitting the development of therapeutic antibodies and cancer vaccines. Cancer immunotherapy was chosen as the ‘*advance of the year*’ for 2016 by the American Society of Clinical Oncology (ASCO) because ‘[i]*n just a few short years, researchers and regulators have moved several different immunotherapy strategies from bench to bedside*’ [7]. Highlighted in the ASCO report are the monoclonal antibody drugs ipilimumab, nivolumab and pembrolizumab, which function as immune checkpoint inhibitors that enhance the body’s immune response to tumors, as well as novel approaches to boost the ability of T cells to detect and destroy cancer. One of these approaches involves the administration of a therapeutic cancer vaccine that delivers tumor antigen, with the aim of priming and activating tumor-specific T cells.

The field of vaccine-based immunotherapy is soon to receive an enormous boost by Cancer MoonShot 2020, a coalition of biotech and pharmaceutical companies, medical universities, and other institutions in the United States (US), sponsored and funded by the US government [8]. The project aims to drive drug discovery by enrolling 20,000 patients with 20 tumor types in clinical trials, with the goal of developing effective cancer cures by 2020. Proponents of the program argue that a well-funded effort will succeed in its mission, just as the US succeeded in sending astronauts to the Moon in 1969. Others are more cautious, arguing that eliminating cancer is more complex than space travel, because cancer is not a single disease and individual tumors are not homogeneous entities [9].

The complexity of cancer is ever more apparent with every new discovery. Genome-wide association studies have shown that only a small fraction of an individual’s risk for cancer can be predicted by their genetic constitution, and that hundreds of genetic variants conspire to determine that risk [10]. Often, disease-related genetic variants do not alter protein-coding regions of the genome, and evidence is emerging to show that they influence cell physiology by altering non-coding RNAs with gene-regulatory roles [11]. Additional layers of complexity have emerged from the sequencing of cancer genomes. These efforts have revealed large intra-individual heterogeneity in neoplasms of the same organ and histotype, i.e., each tumor has its own mutational profile (for lung cancer, see [12]). Additionally, they have uncovered substantial intra-tumoral heterogeneity that complicates treatment decisions and calls into question the strategy of genotyping tumoral DNA using a single biopsy [13]. Altogether, this new understanding of cancer complexity is the driving force in the development of diagnostic tests for the molecular profiling of tumors, which may guide the choice of suitable personalized therapies for each patient.

This Forum article illustrates how these efforts in translational oncology are unraveling in five major cancers – colorectal, ovarian, breast, lung, and urological cancers. In each section, the authors highlight what they see as the most important breakthrough from translational oncology in their field.

- Antoni Castells reports on colorectal cancer (CRC), highlighting how gene expression profiling allowed the identification of four molecular subtypes, one of which is susceptible to immunotherapy with pembrolizumab; molecular profiling in this cancer also facilitated the development of a diagnostic test that detects tumor DNA in stool.
- Vathany Kulasingam and Eleftherios P. Diamandis discuss a large, longitudinal United Kingdom screening program for ovarian cancer (OvCa) involving serum measurements of a tumor biomarker, namely carbohydrate antigen 125; although the cost-effectiveness of this program is yet to be defined, the program created a biobank that will support translational research from the clinic back to the laboratory.
- Helena Earl reports on breast cancer, pointing to the many biomarkers already used to inform therapeutic decision-making and highlighting “liquid biopsy” (i.e., assay for circulating tumor DNA in blood) as a non-invasive way to monitor changes in a tumor’s genetic profile and response to therapy.
- Wade T. Iams and Christine M. Lovly report recent advances in lung cancer research, focusing on oncogene-driven targeted therapy and immune checkpoint inhibitors that have changed the survival expectations for patients with advanced disease.
- J.P. Michiel Sedelaar and Jack A. Schalken discuss recent milestones in prostate cancer research, highlighting how whole exome and transcriptome sequencing of biopsy specimens generated “clinically actionable information” about genomic aberrations in the majority of investigated patients.

Overall, this Forum highlights the state of the art of translational research in oncology, demonstrating several clinical successes but also disclosing areas in which further in-depth bench-to-bedside-and-back research is needed to increase the accuracy of early diagnoses, reduce therapy- and disease-related side effects, and improve the quality of life of patients during treatment.

## Translational advances in colorectal cancer (CRC) research

**Fig. 2:**
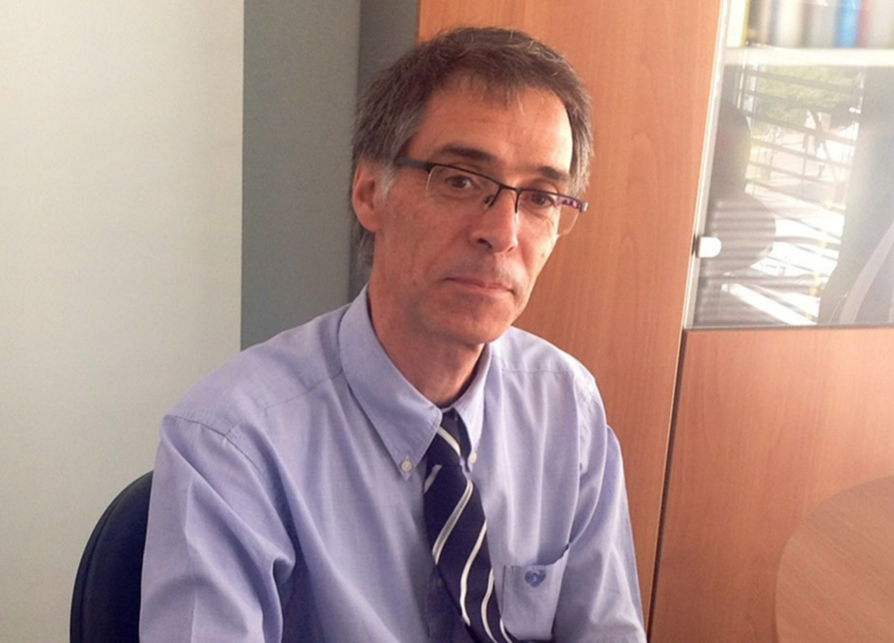
Antoni Castells. Antoni Castells is Associate Professor at the University of Barcelona, School of Medicine. He is a specialist in gastroenterology, and since 2016, has been Medical Director of the Hospital Clinic of Barcelona. His professional activity has been related to colorectal cancer, involving its diagnosis, therapeutics and prevention, being Coordinator of the Colorectal Cancer Unit and Co-coordinator of the Barcelona Colorectal Cancer Screening Program. His research achievements are mainly derived from two internationally-recognized studies, the EPICOLON and COLONPREV projects, in which he is leading principal investigator

Antoni Castells (Fig. 2)

CRC is one of the most prevalent cancers in Western countries and the second cause of cancer-related deaths worldwide [14], both of which justify considering CRC a major healthcare problem. However, from an academic perspective, its relevance goes beyond these figures, as it is a paradigm of translational oncology. Indeed, there are very few neoplasms, if any, in which research has been translated into clinical practice so rapidly and efficiently, thus continuously modifying our approach to the diagnosis, treatment and prevention of the disease.

In the last few decades, tremendous advances have been achieved in the characterization of most molecular mechanisms involved in colorectal carcinogenesis. Indeed, three pathways of genetic instability, namely chromosomal instability, microsatellite instability (MSI), and methylation, have been identified as being responsible for both sporadic and inherited CRC forms [15, 16]. Although several key investigations had uncovered some of the critical genes implicated in these pathways (i.e., *APC*, *KRAS*, *TGF-β*, *TP53*, *PIK3CA*, and DNA mismatch-repair (MMR) genes), a fully integrated view of the genetic and genomic changes and their significance for colorectal tumorigenesis was lacking until the Cancer Genome Atlas project reported a comprehensive molecular view [17]. This extraordinary effort, using different genome-scale approaches (i.e., exome sequence, DNA copy number, promoter methylation, and messenger RNA and microRNA expression), has contributed to the classification of 15 % of these tumors as hypermutated (three-quarters of them exhibiting MSI, usually with hypermethylation and *MLH1* silencing, and one-quarter with somatic MMR gene and *POLE* mutations) [17]. Excluding these hypermutated cases, CRC tumors were found to have considerably similar patterns of genomic alteration; 24 genes were significantly mutated and, in addition to those in the expected *APC*, *TP53*, *SMAD4*, *PIK3CA*, and *KRAS* genes, other frequent mutations were observed in *ARID1A*, *SOX9* and *FAM123B* [17]. There is little doubt that results of this seminal investigation have enabled a deeper understanding of CRC pathophysiology and will definitely contribute to the identification of new therapeutic targets.

CRC, as other neoplasms, is a disease with heterogeneous outcomes and drug responses [18]. On the other hand, it is widely accepted that gene expression-based subtyping is a valuable approach for patient stratification. In the last few years, different molecular CRC classifications have been proposed with the final goal of advancing towards a more personalized medicine. However, their impact on clinical practice has been scarce, mainly because of their discrepant results. To resolve these inconsistencies and facilitate clinical translation, an international consortium dedicated to large-scale data sharing has made considerable effort to coalesce six independent classification systems into four consensus molecular subtypes with distinguishing features: MSI immune (hypermutated, microsatellite unstable, and strong immune activation), canonical (epithelial, marked WNT and MYC signaling activation), metabolic (epithelial and evident metabolic dysregulation), and mesenchymal (prominent TGF-β activation, stromal invasion and angiogenesis) [19]. This new classification approach based on well-differentiated biological profiles may represent a landmark for future clinical stratification and subtype-based targeted interventions [18].

An example of how advances in basic science are revolutionizing the therapeutic approach to CRC is the recent demonstration of MSI as predictor of a favorable response to immunotherapy in patients with advanced neoplasms [20]. Indeed, these authors hypothesized that tumors with a large number of somatic mutations due to MMR defects (those exhibiting MSI) were susceptible to immune checkpoint blockade. Using pembrolizumab, an anti-programmed death 1 immune checkpoint inhibitor, it has been possible to demonstrate an increased progression-free survival (hazard ratio (HR), 0.10; *P* < 0.001) and overall survival (HR, 0.22; *P* = 0.05) in patients with MMR-deficient CRC with respect to those with MMR-proficient lesions [20]. This beneficial effect was not limited to patients with CRC, but also extended to other MMR-deficient tumors, thus emphasizing the fact that advances in one particular type of cancer can be extrapolated to other neoplasms sharing the same underlying molecular mechanism. It is important to keep in mind, however, that molecular profiling cannot entirely replace conventional tumor classification based on organ site. In fact, a recent study evaluating the usefulness of vemurafenib in patients with various *BRAF* V600-mutated non-melanoma cancers demonstrated that this oncogene seems to be targetable in some, but not all, tumors [21].

Besides targeting therapeutics, molecular profiling may also have potential for CRC screening, a preventive approach to reduce the burden of this disease on healthcare systems. Indeed, the utility of this strategy has been accepted by the European Commission, which encourages the implementation of screening programs throughout Europe [22]. Recommended CRC screening strategies fall in two broad categories: stool tests (i.e., fecal immunochemical testing (FIT) for occult blood) that primarily detect cancer, and structural exams (i.e., flexible sigmoidoscopy, colonoscopy and CT-colonography), which are effective in detecting both cancer and premalignant lesions. Whereas FIT is predominant in Europe and Australia, colonoscopy is the dominant screening modality in the US [23]. Very recently, a new non-invasive, molecular-based strategy has been added to the first category [24]. In this seminal study, a multi-target stool DNA test (which includes quantitative molecular assays for *KRAS* mutations, aberrant *NDRG4* and *BMP3* methylation, and β-actin, as well as a hemoglobin immunoassay) was superior to FIT in terms of sensitivity for detecting CRC (92.3 % vs. 73.8 %, respectively; *P* = 0.002) and, more importantly, advanced precancerous lesions (42.4 % vs. 23.8 %, respectively; *P* < 0.001), albeit at the expense of a lower specificity (89.8 % vs. 96.4 %, respectively; *P* < 0.001) [24].

Finally, an understanding of the genetics of inherited CRC is also important to identify at-risk individuals and to consequently improve diagnostic and therapeutic strategies among gene carriers. Indeed, familial predisposition occurs in approximately 25 % of patients with CRC, being hereditary syndromes with a known genetic defect responsible for up to 5 % of all these tumors [15, 16]. In such a context, genetic testing, thus far, has been performed in a phenotype-driven manner [25]. However, the availability of multigene panels for hereditary cancer risk assessment that allow for next-generation sequencing of numerous genes in parallel is changing the current approach [26]. Although the benefits of such comprehensive testing strategies are still under debate, there are few doubts that they have arrived to stay.

## To screen or not to screen for ovarian cancer (OvCa) – results of UKCTOCS study

**Fig. 3:**
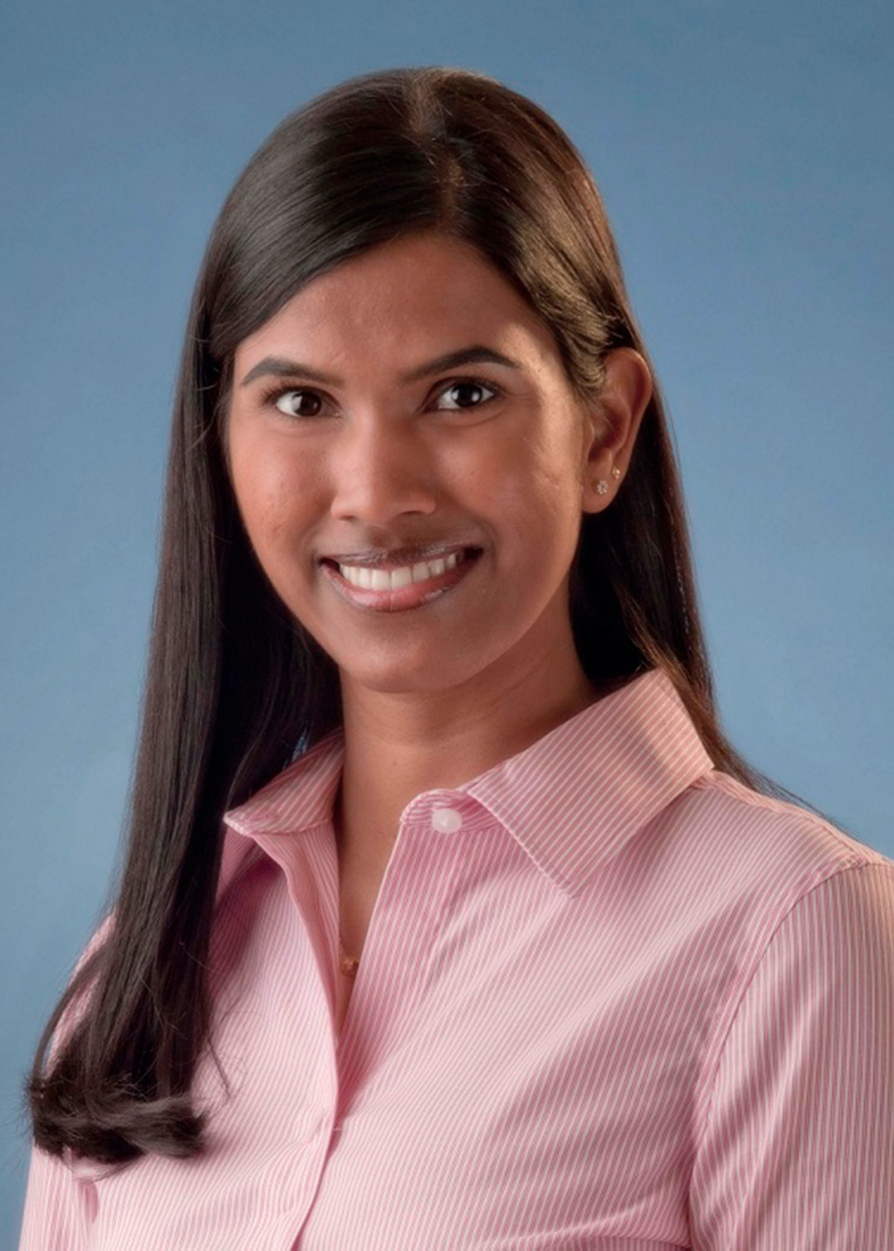
Vathany Kulasingam. Vathany Kulasingam completed her PhD at the Department of Laboratory Medicine and Pathobiology, University of Toronto. Following her PhD, she completed a post-doctoral training diploma program in Clinical Chemistry at the University of Toronto. Dr. Kulasingam is currently a clinical biochemist at the University Health Network in Toronto, an Assistant Professor at the Faculty of Medicine, University of Toronto, and a Fellow of the Canadian Academy of Clinical Biochemistry. Her current interests include novel tumor biomarker discovery and application of proteomics to clinical practice

**Fig. 4:**
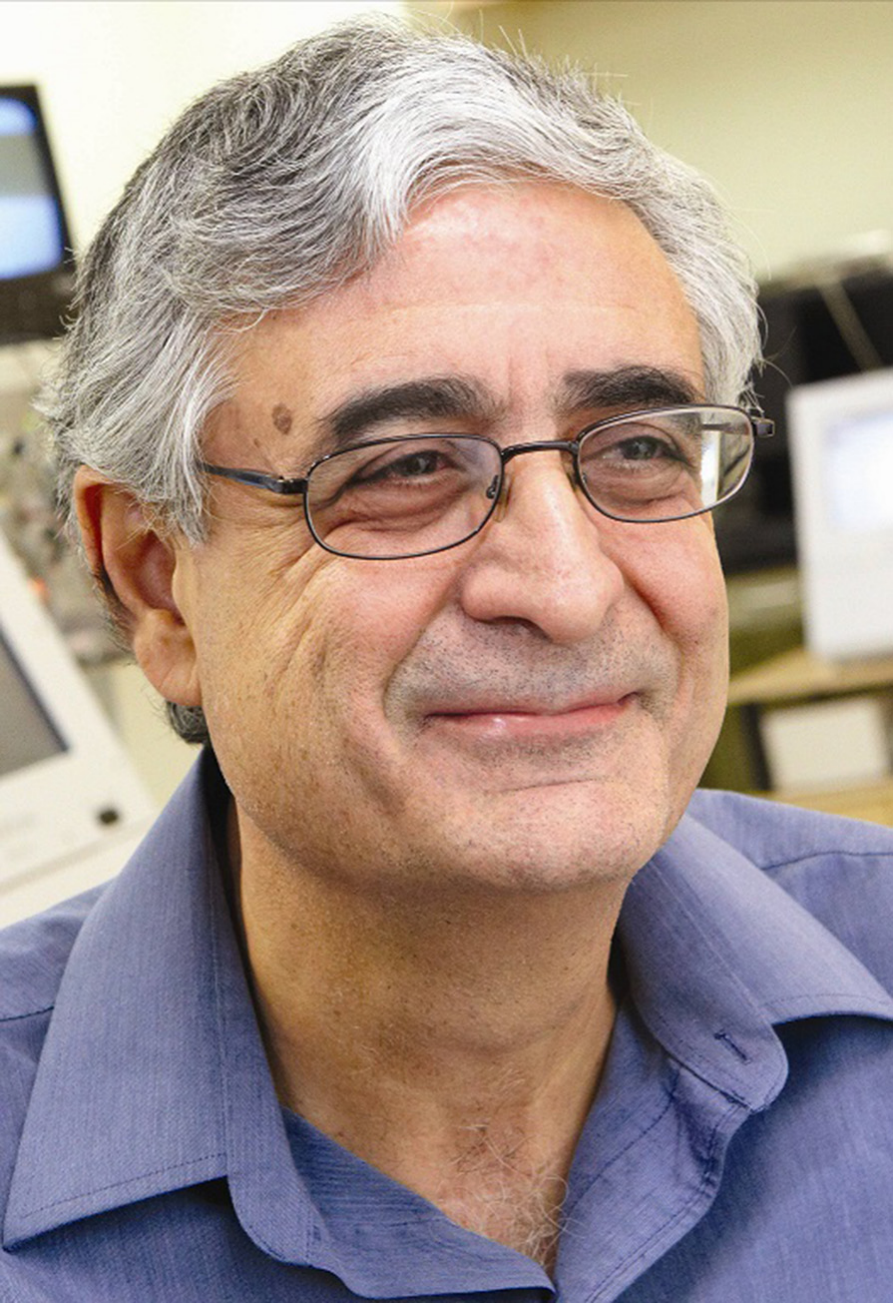
Eleftherios P. Diamandis. Eleftherios P. Diamandis is currently Professor and Head, Division of Clinical Biochemistry, Department of Laboratory Medicine and Pathobiology, University of Toronto, Biochemist-in-Chief at University Health Network, and Division Head of Clinical Biochemistry at Mount Sinai Hospital, Toronto. He is also a “Hold’em for Life” Chair on Prostate Cancer Biomarkers, a Corresponding Member of the Academy of Athens, a Member of the Royal Society of Canada and the Canadian Academy of Health Sciences, and an elected Fellow of the American Association for the Advancement of Science

Vathany Kulasingam and Eleftherios P. Diamandis (Figs. 3 and 4)

OvCa is the most lethal gynecological malignancy, accounting for 5–6 % of all cancer-related deaths. As with any cancer, the notion of earlier OvCa diagnosis and population screening is an attractive but challenging proposition; OvCa has low prevalence (0.1 %), which means that, to be of clinical relevance, a screening test must have a positive predictive value of > 10 %, with a specificity of > 99.6 % and a sensitivity of > 75 %. In addition, since OvCa lacks a recognizable latent phase, overdiagnosis, misdiagnosis, and creation of a false sense of security are some of the potential adverse effects of screening. Using prostate-specific antigen as a screening test for prostate cancer as an example, two large randomized prospective trials have been completed and it is still unclear whether the benefits outweigh the harms in this setting [27]. On the contrary, screening for cervical cancers has resulted in a significant (50–90 %) reduction in disease-specific mortality.

Based on the World Health Organization’s 10 principles of screening [28] (including, but not limited to, the disease being an important health problem as well as appropriate treatment and a suitable test acceptable to the population being available), population screening for OvCa is warranted. The ability to detect human malignancy via a simple blood test has long been an objective in medical screening. The advantages of such an easy to use, relatively non-invasive and operator-independent test are self-evident. A variety of ovarian tumor markers have been studied; the most extensively investigated of these being carbohydrate antigen 125 (CA125), a large glycoprotein of unknown function. Since its discovery in 1981 by Bast, Jr. et al [29], CA125 still remains the gold-standard serum biomarker for OvCa. As such, a handful of OvCa screening trials have been undertaken, including the ovarian arm of the Prostate, Lung, Colorectal, Ovarian (PLCO) Cancer Screening trial (USA) [30]. The PLCO trial was a randomized controlled trial (RCT) of approximately 68,000 post-menopausal women, of whom approximately 30,000 underwent screening between 1993 and 2007. The women were screened using serum CA125 with a single cut-off of ≥ 35 kU/L and transvaginal ultrasound for 4 years followed by CA125 alone for a further 2 years. The results showed no reduction in mortality.

However, by far the largest RCT has been the UK Collaborative Trial of Ovarian Cancer Screening (UKCTOCS), whose primary objective was to assess the effect of screening on disease mortality [31]. We believe that this effort, undertaken by Jacob et al., represents one of the most important translational advances in OvCa research. Over 1.2 million women were recruited from 13 regional centers in England, Wales and Northern Ireland over the course of approximately 4 years, starting in 2001, with approximately 200,000 post-menopausal women being randomly assigned in a 1:1:2 ratio to annual multimodal screening (MMS) with serum CA125 interpreted with the risk of OvCa algorithm (ROCA) and with transvaginal ultrasound (USS), annual USS alone, or no screening. The study was powered to detect a mortality reduction of 30 %. At a median 11 years’ follow-up, OvCa was diagnosed in 0.7 % of women in the MMS group versus 0.6 % in each of the USS and no screening groups. The primary outcome analysis spanning 0–14 years showed no significant reduction in mortality in the MMS and USS groups (15 % and 11 %) when compared to the no screening arm. Nonetheless, a secondary sub-group analysis did show the benefit of screening in women between the latter half of the screening period (years 7–14), when prevalent cases were excluded (28 % mortality reduction after 7 years of screening in the MMS group). The authors state that this is due to the late effect on mortality that is seen in screening trials and that additional follow-up of the UKCTOCS cohort is necessary before “firm conclusions” can be reached on the efficacy and cost-effectiveness of OvCa screening.

One key difference between the UKCTOCS study and other OvCa screening trials is in the interpretation of serum CA125. UKCTOCS used ROCA – an algorithm that compares the CA125 profile (not just a single cut-off value) of cases to that of healthy women and incorporates age-specific incidence of OvCa in calculating risk [32]. Using ROCA, the study triaged women to normal (annual screening), intermediate (repeat CA125 in 3 months), and elevated (repeat CA125 and perform USS). However, a post-hoc analysis of the PLCO cohort using ROCA as the screening modality showed that it would not have produced a statistically significant mortality reduction effect [33].

Despite the modest reduction in mortality in the UKCTOCS trial, the authors have to be given credit for conducting a large scale RCT of this magnitude over the course of approximately 15 years. More than 200,000 participants, > 650,000 annual screening events, a multicenter setting, and strong quality assurance programs for standardization of ultrasound findings are some of the highlights of the RCT study design. Further, the fact that they were able to achieve 99 % compliance on follow-up certainly deserves applause. In addition, a side, and arguably one of the most important, benefits to arise from this exercise is the biorepository they now possess – longitudinal samples with excellent, linked clinical data that can serve as a useful resource for a broad research community to investigate the prevention, early detection, etiology, and treatment of OvCa. Additional follow-up ancillary studies and evaluation of the cohorts with different cut-off values or groups may also be examined, ultimately serving as a rich data-mining resource.

## Translational breast cancer research – past success and future promise

**Fig. 5:**
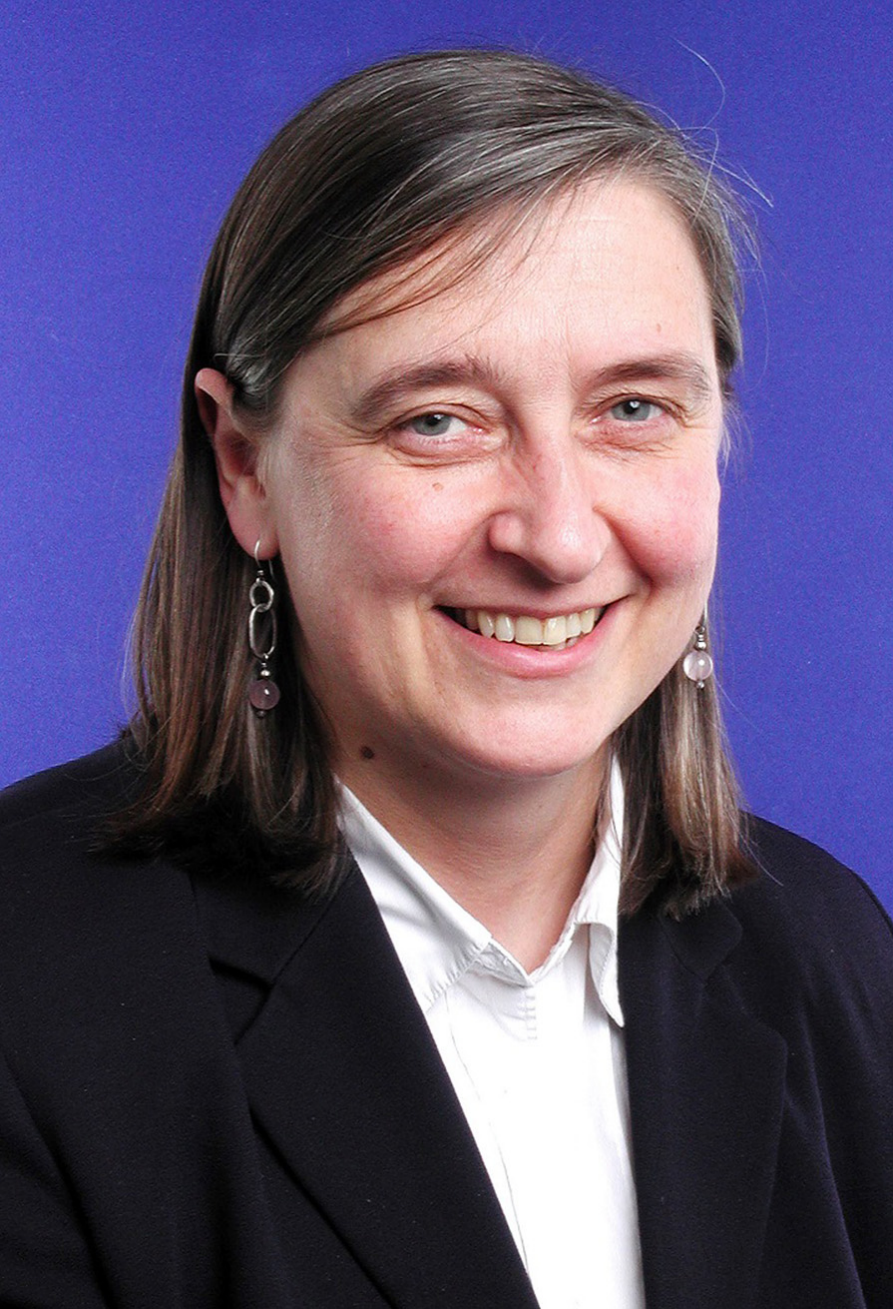
Helena Earl. Helena Earl is an academic clinician in Medical Oncology and currently Professor of Clinical Cancer Medicine at the University of Cambridge, Department of Oncology and a Principal Investigator of the NIHR Cambridge Biomedical Research Centre and Cambridge Experimental Cancer Medicine Centre. She is co-lead for the Breast Cancer Programme at the Cancer Research UK Cambridge Cancer Centre and significantly contributes to the translational endeavor in precision medicine and the development of personalized treatment pathways in breast cancer. In Cambridge, she is the cancer lead in the collaborative workstream for novel adaptive trial designs

Helena Earl (Fig. 5)

Translational cancer research provides a “bridge” between fundamental scientific research and clinical research, often referred to colloquially as “bench-to-bedside”. Scientific cancer researchers, on the one hand, and clinical researchers, on the other, pursue their academic tracks with passion and precision, each in their own increasingly complex environment. So what of the “translational research” bridge? To follow the analogy, a physical bridge is designed by a team of highly trained structural engineers, to last for centuries carrying constant heavy traffic. The equivalent translational cancer research structure, at present, would be more equivalent to a high-wire act or, at best, a single track, one-way, wobbly construction, suspended between mountain tops! It is time to construct a robust, state-of-the-art translational bridge, built to last.

The cultural challenges in bringing together the scientific and clinical research groups in a meaningful dialogue are considerable; there needs to be mutual understanding and respect across the bridge, which is sensitive to the endeavors on both sides. The recent journal debate around more public access to clinical trials data has been timely [34]. Clinical trials data, painstakingly collected and analyzed, clearly need to be published fully, including that of negative or inconclusive studies. Nevertheless, the high quality clinical data from trials should remain the asset of researchers who have devoted their careers to developing, running, and analyzing these clinical trials. Clearly, with appropriate safeguards, these data can be shared, along with the translational samples being increasingly collected within large phase 3 clinical trials [35]. Thus, a sensitive partnership can be developed to move forward – without the clinical research effort in trials, there would be less high-quality data, with allied translational tumor and germline banks for scientists to investigate.

So, in the translational effort, could large clinical trials be replaced by ‘big data’ national and international collections from breast cancer populations? Whilst the short answer is ‘yes’, tumor and germline cancer genomics collections are now being developed, and will enable translational research to be carried out routinely within cancer centres. Undoubtedly, the advantages of clinical trial-based translational research will remain. These include more homogeneous populations of patients, treated in a standard way, with excellent governance of treatment delivery, patient safety, follow-up, and short- and long-term outcomes analyses. Routine data collection in cancer clinics/centres will have to become an order of magnitude better to replace the data available within clinical trials, and apart from other challenges, will incur considerable cost.

Translational breast cancer research includes past success and enormous future potential and has been at the forefront of this field. The ultimate goal is the application of precision medicine and personalized breast cancer care to all patients. Notable successes include biomarkers of treatment response in routine clinical use such as ER, PR, HER2, and Ki67. A large number of predictive biomarkers of response to targeted, novel agents are emerging, although there remain considerable challenges in terms of the robustness of these in the clinic. Many large phase 3 breast cancer clinical trials have translational collections that have been utilized for translational research in terms of tumor biomarkers [35], germline predisposition to breast cancer and association with prognosis [36], and pharmacogenetics [37]. Phase 2 breast cancer trials have embedded a rich seam of translational research and, in this context, ‘window’ studies carried out pre-operatively are being utilized [38]. The neoadjuvant context has been a translational treasure trove, likely to lead to the more rapid introduction of novel therapies with significant translational discovery [39]. Breast cancer researchers have led the field with the discovery and validation of tumor genomic stratification [40, 41] and plasma circulating tumor DNA (ctDNA) in both metastatic [42] and early breast cancer [43].

There are two recent advances in breast cancer translational research that deserve particular mention. The first is the outstanding work of the METABRIC (Molecular Taxonomy of Breast Cancer International) Consortium [40], which was the first to discover and validate 10 clusters of breast cancer stratified by long-term breast cancer-specific survival, an important recent advance in translational breast cancer research. This analysis was carried out on 2000 breast cancers, collected in fresh tissue tumor banks and funded within an international consortium. The same group has recently refined this dataset with the definition of somatic tumor mutations in a total of 2500 fresh breast tumors [41]. This detailed translational research will inform not only breast cancer prognosis within the 10 groups, but also the underlying biology of these novel stratifications of breast cancer. In addition, this will allow the potential to utilize these to predict response not only to novel targeted small molecule therapies, but also an increasing number of novel immune therapies through both antibody and vaccine treatments. These novel stratified groups have been defined from the start with access to the clinical follow-up data and, inevitably, the advances achieved will have important impact and treatment outcomes on patients with breast cancer.

The second is the use of ctDNA to track mutations and response to treatment in both metastatic [42] and neo/adjuvant breast cancer [43]. The forefront translational research in Cambridge, UK has been important to define and integrate ctDNA into clinical/translational research in metastatic breast cancer. Following the original descriptions of rapid biomarker response to treatment defined in the seminal publication [41], ctDNA is now being embedded into study designs. Further elegant research demonstrates the ability to track specific mutations present in the primary tumor [43], which now offers the potential to include ctDNA tracking into the design of novel feasibility studies due to start in 2016. In the near future, the use of ctDNA as a repeatable “liquid biopsy” will become a reality, allowing translational and clinical researchers the potential of a dynamic tracking of tumor heterogeneity and the distinct possibility of personalizing precision treatments for patients in real time.

## Recent advances in translational lung cancer research

**Fig. 6:**
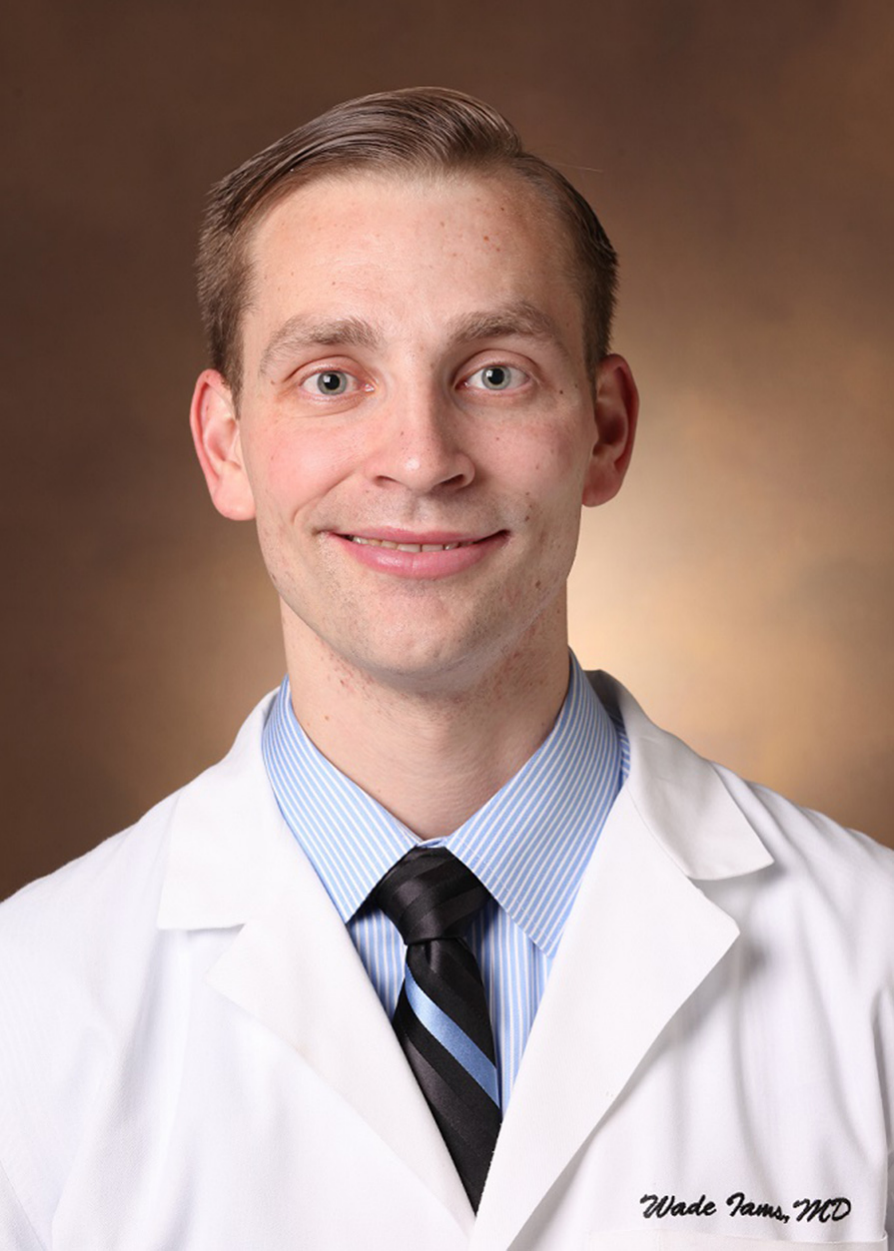
Wade Iams. Wade Iams has recently completed his Chief Residency at Vanderbilt University Medical Center, and is beginning his clinical fellowship in hematology/oncology at McGaw Medical Center of Northwestern University. He is an active member of the American Society of Clinical Oncology (ASCO), International Association of Lung Cancer Research (IASLC), and the American Association for Cancer Research (AACR), where he also serves on the Associate Member Fundraising Committee. He has been awarded a Young Investigator Award from Adaptive Biotechnologies as well as travel awards from the AACR and IASLC for early-career research on thoracic malignancies

**Fig. 7:**
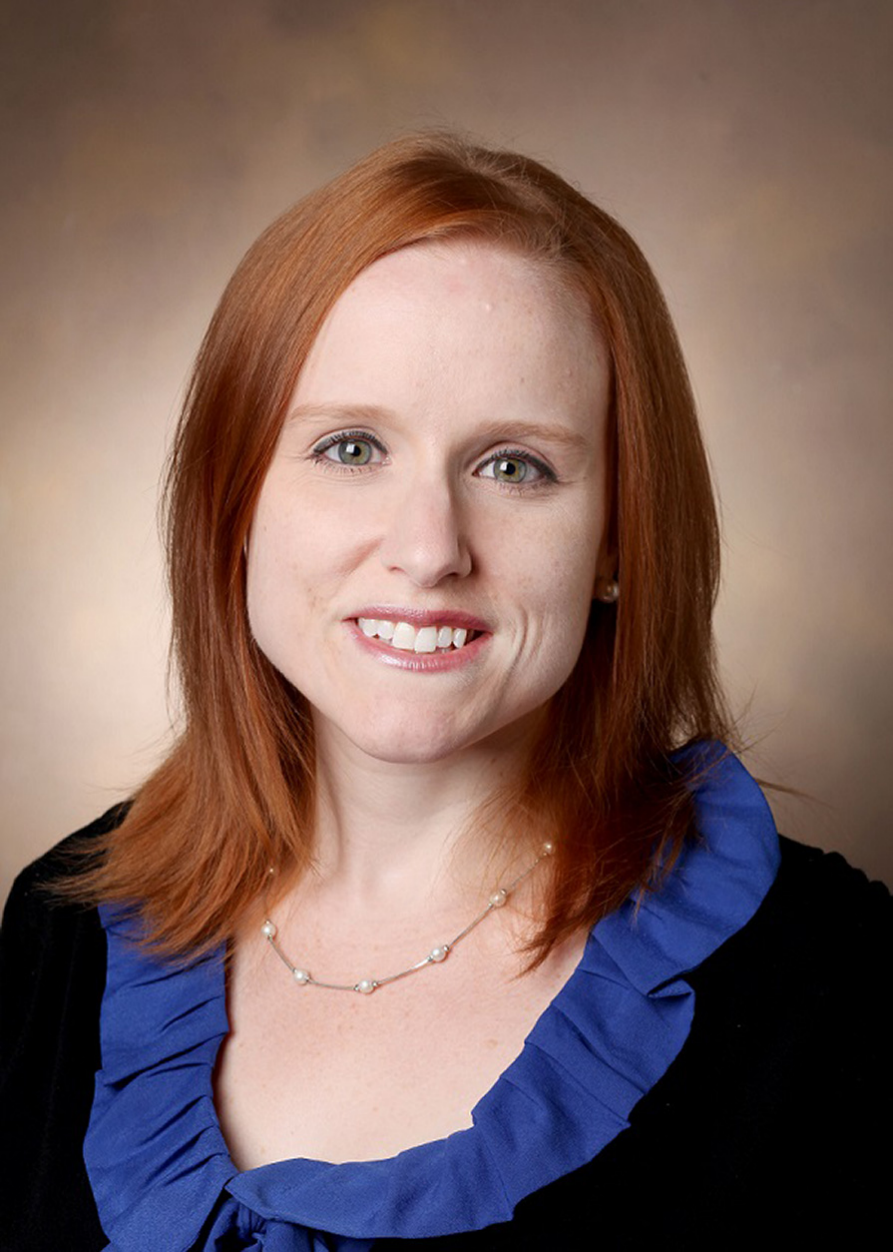
Christine M. Lovly. Christine M. Lovly is an Assistant Professor of Medicine and Cancer Biology at Vanderbilt University Medical Center. Dr. Lovly is a physician-scientist with a special interest in thoracic malignancies. Her laboratory research is directed at understanding and developing improved therapeutic strategies for specific clinically relevant molecular subsets of lung cancer. Dr. Lovly has received independent grant funding from several foundations, including Uniting Against Lung Cancer, Conquer Cancer Foundation, LUNGevity, Damon Runyon Foundation, and V Foundation. She is an active member of the American Society of Clinical Oncology (ASCO), the International Association for the Study of Lung Cancer (IASLC), and the American Association for Cancer Research (AACR). She is also co-editor-in-chief for the website www.mycancergenome.org, a Vanderbilt initiated, freely available website which aims to provide health care practitioners, patients and advocates with up-to-date information regarding genetically informed cancer medicine

Wade T. Iams and Christine M. Lovly (Figs. 6 and 7)

Lung cancer is the leading cause of cancer deaths worldwide [44]. More than 85 % of lung cancers are classified as non-small cell lung cancer (NSCLC), as defined by histologic features. NSCLC can be divided into several subtypes, of which adenocarcinoma is the most common, comprising over 50 % of all lung cancers [45]. Although histological features currently remain the basis of clinical diagnosis, advances in molecular oncology have allowed researchers to identify oncogenes and therapeutic targets in NSCLC such as epidermal growth factor receptor (*EGFR*). Tremendous progress has been made in advancing the diagnosis and treatment of patients with lung cancer. The progress in translational lung cancer research over the past 5 years can be divided into four broad categories: (1) new treatments for patients with acquired resistance to molecular-targeted therapy; (2) ongoing development of ctDNA (also known as “liquid biopsy”) technology for clinical use; (3) a dramatic surge in the number of clinical trials involving immune checkpoint inhibitors; and (4) early progress in novel therapies for patients with small cell lung cancer (SCLC). We will highlight demonstrative examples from each of these areas of translational research in lung cancer to illustrate the direction of the field.

On November 13, 2015, the United States Food and Drug Administration (FDA) granted accelerated approval to osimertinib for the treatment of patients with NSCLC harboring an EGFR T790M mutation. Mutations in *EGFR* are detected in approximately 30–40 % of Asian patients and 10 % of Caucasian patients with NSCLC [46]. While initial therapy targeting this mutation is quite effective, drug resistance typically develops within 1–2 years [46]. The *EGFR* T790M “second site” mutation is the most common resistance mechanism, affecting 60 % of patients with *EGFR*-mutant NSCLC that has acquired resistance to *EGFR*-targeted therapy [46]. Osimertinib is the first FDA-approved drug for patients with acquired resistance to the FDA-approved EGFR tyrosine kinase inhibitors erlotinib, gefitinib and afatinib. Whereas erlotinib, gefitinib and afatinib were designed against wild-type EGFR, osimertinib was specifically designed to be selective for mutant EGFR, thereby increasing the precision of our therapies [46]. The approval of this drug demonstrates that targeting resistance mechanisms is a successful paradigm for ongoing research to improve outcomes in lung cancer patients with mutant *EGFR* and other known oncogenes, such as the *ALK* and *ROS1* genes.

As the monitoring of dynamic changes in oncogene mutations takes on greater therapeutic importance, developing technology to screen patients for these changes without repeating invasive tumor biopsies is critical. Accordingly, on the same day that osimertinib’s FDA approval was announced, the FDA also announced approval of the first companion diagnostic test to detect *EGFR* T790M mutations in blood (Cobas *EGFR* mutation test v2); this is one example of many assays designed and optimized to detect ctDNA in plasma. The clinical application of this technology represents the progress of nearly a decade of research into strategies to monitor specific lung cancer mutations in the bloodstream and, in some cases, bloodstream mutation monitoring has detected resistance mutations up to 4 months prior to radiographic progression on oncogene-targeted therapy [47, 48]. As specific oncogene mutation-directed therapies proliferate, clinically applicable assays to detect these mutations are being developed in lockstep, such that lung cancer care is rapidly becoming not only genomically personalized, but is doing so in a dynamic manner. ctDNA testing has the potential to revolutionize not only how we diagnose and treat lung cancer, but other solid organ tumors as well.

**Fig. 8:**
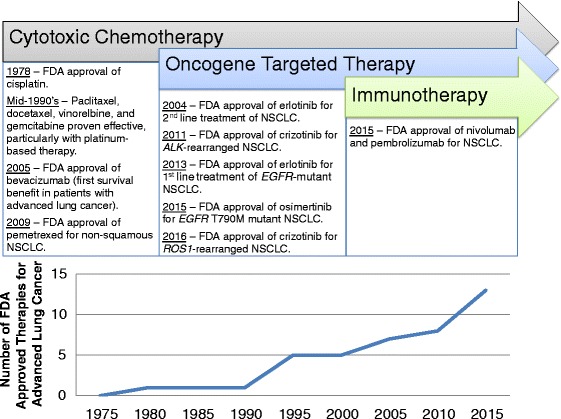
Advances in the treatment of patients with lung cancer over the past 50 years. Advances in the treatment of patients with lung cancer over the past 50 years can be divided into cytotoxic chemotherapy, oncogene-targeted therapy and immunotherapy, with the most recent advances being in oncogene-targeted therapy and immunotherapy. All FDA approvals noted apply to patients with locally advanced or metastatic lung cancer

No discussion of translational research advances in lung cancer would be complete without noting the success and burgeoning clinical trial landscape applying immune checkpoint inhibitors in varying combinations to treat lung cancer patients. Harnessing a patient’s immune system to destroy malignant cells has been a treatment goal for decades [49]. However, not until March 2015 were any specific immune system activators FDA-approved to treat lung cancer (Fig. 8). Currently, two immune checkpoint inhibitors (both inhibitors of programmed death-1) have been approved for the treatment of patients with advanced NSCLC – nivolumab (FDA approved March 4, 2015) and pembrolizumab (FDA approved October 2, 2015) [50]. Many ongoing clinical trials seek to combine immune checkpoint inhibitors with additional immune modulatory agents, chemotherapy or targeted therapy. As a single example, the ongoing CheckMate 227 trial (NCT02477826) [51] will enroll patients with previously untreated advanced NSCLC and stratify them into four arms, namely chemotherapy alone, nivolumab alone, nivolumab combined with ipilimumab (an immune checkpoint inhibitor of cytotoxic T lymphocyte antigen 4), and nivolumab combined with chemotherapy. The combination of different immune checkpoint inhibitors seeks to increase efficacy by addressing more than one pathway through which tumors evade the immune system, and the combination of immune checkpoint inhibitors with chemotherapy seeks to capitalize on the antigenic stimulation of tumor cell apoptosis induced by chemotherapy. As an example of the combination of immune checkpoint inhibitors and oncogene mutation-targeted therapy, the TATTON clinical trial (NCT02143466) assessed the combination of osimertinib and durvalumab (an immune checkpoint inhibitor of programmed death ligand 1) in patients with *EGFR*-mutant NSCLC. This trial sought to target the oncogenic driver mutation in these patients while simultaneously promoting immune recognition during tumor apoptosis. Initial results from this trial have shown an increased incidence of pneumonitis in the combination therapy arm [52]. This example strikes an important cautionary note regarding the potential increased toxicities associated with combining different agents. These adverse events, as well as the so-called “financial toxicity” of these very expensive therapies (for example, using nivolumab to treat metastatic renal cell carcinoma has been shown to cost patients and insurers at least $65,000 [53]), bring to the forefront the need to rationally design clinical trials by understanding the molecular mechanisms underpinning response or resistance to these agents. Optimal translational research using clinical trials data will most safely and efficiently advance the field of implementation of these new treatment modalities.

While NSCLC is the most common form of lung cancer, 15 % of patients with lung cancer have SCLC. Patients with this disease have not seen a change in therapy for over 30 years [54]. The success of nivolumab and pembrolizumab in patients with NSCLC has spurred clinical trials of each agent in patients with SCLC (NCT01928394 and NCT02054806, respectively). Not only immune checkpoint inhibitors, but molecularly targeted therapies are being assessed in clinical trials in patients with SCLC. Delta-like protein 3 is an inhibitor of neuroendocrine cell differentiation preferentially expressed in patients with SCLC compared to control patients [55]. An inhibitor of Delta-like protein 3, Rovalpituzumab Tesirine, is being tested in an ongoing clinical trial (NCT02674568). These clinical trials demonstrate the potential for treatment improvements in the near future for patients with this recalcitrant malignancy.

While lung cancer remains the most lethal malignancy worldwide, the success of oncogene-driven targeted therapy and immune checkpoint inhibitors has already changed the landscape of survival for patients with advanced disease. As the aforementioned translational research advances indicate, the field is rapidly progressing through paradigm shifting techniques; physicians should be aware of the recent improvements in outcomes for patients with this disease.

## Recent breakthroughs in translational prostate cancer research

**Fig. 9:**
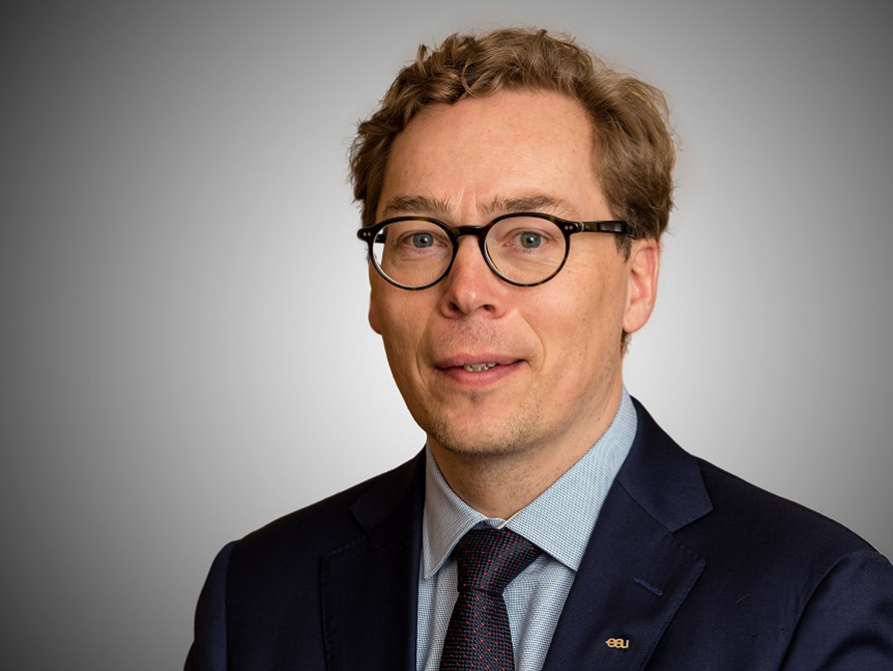
J.P. Michiel Sedelaar. J.P. Michiel Sedelaar is the deputy-chief of the department of Urology at Radboud University Medical Center, Nijmegen, the Netherlands. He finished his urology training in 2007 and in 2008–2009 he was a post-doc researcher at the Johns Hopkins Medical Institutes in Baltimore, MD, USA. His focus during that time was the development of smart-drugs for detection and treatment of prostate cancer and steroidogenesis of prostate cancer. Since 2010, he returned to Nijmegen for an onco-urology staff position. His research achievements are mainly on imaging studies for prostate cancer (use of MRI, MRI-guided treatments) and basic research on the androgen receptor in prostate cancer. He has national and international board functions; among others, he is the chair of the EAU-Young Urology Office

**Fig. 10:**
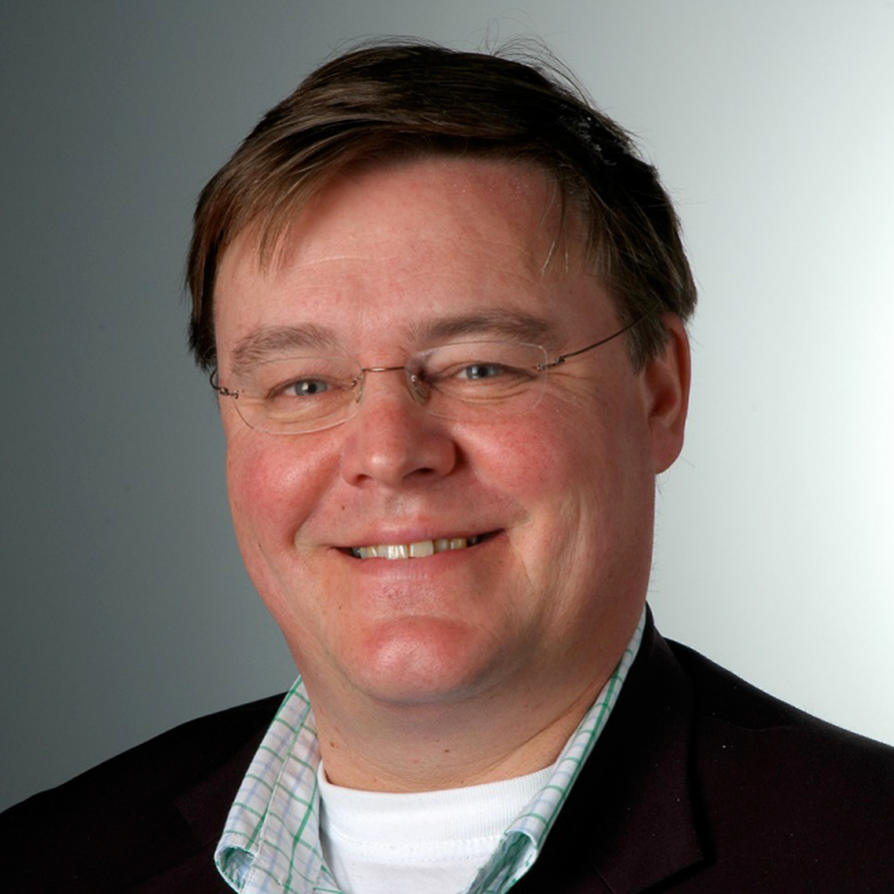
Jack A. Schalken. Jack A. Schalken trained as biochemist at Radboud University Medical Center, Nijmegen, the Netherlands, where he is Director of the urology research lab. His work focusses on translational research, with an emphasis on biomarkers and novel therapeutic targets such as epithelial–mesenchymal transition. His team’s research led to the clinical introduction of the first urine-based prostate cancer test (Progensa PCA3, 2006) and more recently to the SelectMdX test, which has been commercially available for clinical use since 2015. In 1996, he was appointed as full professor at the University of Utrecht (experimental oncology), and in 2001 as professor of experimental urology (Radboud University Medical Center). He has been awarded several scientific awards, most recently the Dominique Chopin award (2015) for his long-term contribution to the field

J.P. Michiel Sedelaar and Jack A. Schalken (Figs. 9 and 10)

With the abundance of scientific work in the field of oncological urology, it is very hard to distinguish the most important recent translational advances in urological cancer research. When do you consider science to be a real clinical game changer, in comparison to basic research work which in itself explains small steps in an oncological phenomenon, but in fact can lead (by serendipity sometimes) to breakthroughs? Even the fact that a paper may be considered a milestone is often subject to a difference of opinions. Nevertheless, we highlight herein four papers that, in our opinion, represent groundbreaking advancements in translational research. One of our major considerations for candidates for these milestones in translational research would be the immediate impact on the direct translation of the presented findings into clinical practice. The selected papers were published in high impact journals within the last 18 months. Non-surprisingly, all papers are the fruit of labor from well-established international and multidisciplinary research teams.

The first paper to be discussed as a game changer in the field of personalized prostate cancer care is that by Robinson et al. [56]. The group presents their work on a multi-institutional clinical sequencing infrastructure to conduct prospective whole-exome and transcriptome sequencing of bone or soft tissue tumor biopsies from a cohort of 150 castration-resistant prostate cancer (CRPC)-affected individuals. CRPC is the terminology used for patients with prostate cancer who progress despite the use of hormonal treatment. Even with the combination of luteinizing hormone releasing hormone analogs and the anti-androgen blockade, patients progress on prostate-specific antigen (PSA) level, symptoms and/or imaging. In these patients, the androgen receptor (AR) is still a major driver of prostate cancer. The authors describe several known and new aberrations in the genome. Aberrations of AR ETS genes, *TP53* and *PTEN*, were frequent, with, not surprisingly, enriched *TP53* and *AR* alterations in metastatic CRPC compared to primary prostate cancer. New genomic alterations were found in *PIK3CA/B*, R-spondin, *BRAF/RAF1*, *APC*, β-catenin, and *ZBTB16/PLFZ*. Aberrations in *BRCA2*, *BRCA1* and *ATM* were observed at substantially higher frequencies compared to those in primary prostate cancers. Of all patients, 89 % harbored a clinically actionable aberration, mostly in the AR (62.7 %) and in other cancer-related genes (65 %), and 8 % with actionable pathogenic germline alterations. This multi-institutional group concluded that the cohort study provides the clinically actionable information that could impact treatment decisions for these patients. The findings underline the fact that CRPC is a general term used for an accumulation of many different types of genomic alterations in prostate cancer, which should all be treated differently. This study provides prostate cancer clinicians grounds for re-biopsy of their patients during the course of the disease and treatment, in order to facilitate personalized medical treatment for their developing CRPC.

The next groundbreaking paper is that by Mateo et al. [57], which indicates that the model proposed, i.e., precision medicine based on the molecular profile of the cancer’s actionable targets, can be validated. This study describes a phase 2 trial conducted in patients with progressive metastatic CRPC (mCRCP) and treated with olaparib. Olaparib is a new poly(adenosine diphosphate [ADP]-ribose) polymerase (PARP) inhibitor. PARP is involved in multiple aspects of DNA-repair and the PARP-inhibitor olaparib has been approved for the treatment of OvCa with *BRCA1/2* mutations. PARP-inhibition has durable antitumor activity in men with mCRPC and deleterious germline *BRCA2* mutations, a disease subset of mCRPC associated with a poor prognosis [58]. Fifty eligible patients had histologically confirmed mCRPC with progression after chemotherapy. All patients were treated with 2dd 400 mg olaparib. The primary endpoint was the response rate, defined as a response according to RECIST criteria, or a PSA-decrease of 50 % or more, or a conversion in the circulating tumor-cell count from > 5/7.5 mL to < 5/7.5 mL. For all patients, biomarker studies were prospectively planned. Of the 49 evaluated patients, 16 had a response to olaparib with a median duration of treatment of 40 weeks. Overall, 16 patients (33 %) had tumor aberrations in DNA-repair genes. Patients with aberrations in DNA-repair genes had a significantly higher response to olaparib. Specifically, the seven patients with *BRCA2*-loss had PSA levels that fell by 50 % or more from baseline. From these seven patients, the five with measurable disease also had a radiologic partial response. Further, four out of five patients with deleterious *ATM* mutations had a response to olaparib. The authors concluded that the results of this phase 2 clinical trial suggest that a common subset of metastatic prostate cancers can be molecularly stratified for treatment using high-throughput next generation sequencing assays. Very much like Robinson et al. [56], this paper underlines the need for re-biopsy of patients during the course of their disease in order to achieve personalized treatment.

The last two breakthrough papers are discussed together, since these have been published by the same research group. Gao et al. [59] and Drost et al. [60] respond to the lack of in vitro prostate cancer models that reflect the diversity in human prostate cancer. The authors describe the development of a 3D-organoid system for long-term individual culture of prostate cancer from biopsy specimens and circulating tumor cells. The methodology described in these manuscripts should enable the generation of a large repertoire of patient-derived prostate cancer-lines amenable to genetic and pharmacologic studies. As the authors explain, the past few years have seen a dramatic increase in the number of approved therapies, with several promising investigational agents in late stage development for mCRPC. The response to these novel agents, however, is highly variable and only about 30 % of mCRPC patients will attain a durable response of more than 6 months. These models can be used to study the ever growing number of approved therapies for mCRPC, the combination and sequencing of such therapies, and to predict patient response.

As stated above, the process of selecting game changing papers from such an enormous field of translational prostate cancer studies will lead to other research milestones being ignored. Nevertheless, aside from personal opinion, the selected papers have also been recognized by the research community as being groundbreaking. Their common denominator is the acknowledgement of the diversity of genomic alterations in mCRPC and the need for a personalized treatment option for these patients. Using fresh tissue re-biopsy, circulating tumor cells or 3D-organoid cell systems, the genomics of mCRPC can be studied and responses to different therapies could even be predicted. These studies have most definitely changed the understanding of mCRPC and the methods through which the ever expanding armamentarium of prostate cancer treatments are selected for individual patients, thus improving patient outcomes.

## Abbreviations

AR, androgen receptor; CA125, carbohydrate antigen 125; CRC, colorectal cancer; CRPC, castration resistant prostate cancer; ctDNA, circulating tumor DNA; EGFR, epidermal growth factor receptor; FDA, United States Food and Drug Administration; FIT, fecal immunochemical testing; mCRPC, metastatic castration resistant prostate cancer; MMR, mismatch-repair; MMS, multimodal screening; MSI, microsatellite instability; NSCLC, non-small cell lung cancer; OvCa, ovarian cancer; PLCO, Prostate, Lung, Colorectal, Ovarian; PSA, prostate-specific antigen; RCT, randomized controlled trial; ROCA, risk of ovarian cancer algorithm; SCLC, small cell lung cancer; UKCTOCS, UK Collaborative Trial of Ovarian Cancer Screening; USS, transvaginal ultrasound
